# Correlates of health information seeking between adults diagnosed with and without cancer

**DOI:** 10.1371/journal.pone.0196446

**Published:** 2018-05-10

**Authors:** Eric Adjei Boakye, Kahee A. Mohammed, Christian J. Geneus, Betelihem B. Tobo, Lorinette S. Wirth, Lei Yang, Nosayaba Osazuwa-Peters

**Affiliations:** 1 Saint Louis University Center for Health Outcomes Research (SLU*COR*), Saint Louis University, Saint Louis, Missouri, United States of America; 2 Department of Environmental and Occupational Health, College for Public Health and Social Justice, Saint Louis University, Saint Louis, Missouri, United States of America; 3 Department of Epidemiology, College for Public Health and Social Justice, Saint Louis University, Saint Louis, Missouri, United States of America; 4 Department of Biostatistics, College for Public Health and Social Justice, Saint Louis University, Saint Louis, Missouri, United States of America; 5 Saint Louis University Cancer Center, Saint Louis, Missouri, United States of America; 6 Department of Otolaryngology-Head and Neck Surgery, Saint Louis University, Saint Louis, Missouri, United States of America; Public Library of Science, UNITED KINGDOM

## Abstract

**Purpose:**

To examine predictors of information seeking behavior among individuals diagnosed with cancer versus those without.

**Methods:**

Cross-sectional data from the Health Information National Trends Survey 4 Cycles 1–3 (October 2011 to November 2013) were analyzed for 10,774 survey respondents aged ≥18 years. Binary logistic regression was used to examine the effect of socio-demographic and behavioral factors on health information seeking.

**Results:**

Cancer diagnosis did not predict health information seeking. However, respondents diagnosed with cancer were more likely to seek health information from a healthcare practitioner. Compared to males, females were more likely to seek health information irrespective of cancer diagnosis. Regardless of cancer diagnosis, those without a regular healthcare provider were less likely to seek health information. Likelihood of seeking health information declined across education strata, and significantly worsened among respondents without high school diplomas irrespective of cancer diagnosis.

**Conclusions:**

Respondents sought health information irrespective of cancer diagnosis. However, the source of health information sought differed by cancer diagnosis. Gender, education, and having a regular healthcare provider were predictors of health information seeking. Future health communication interventions targeting cancer patients and the general public should consider these findings for tailored interventions to achieve optimal results.

## Introduction

The health information landscape of the United States is constantly evolving and proliferating due to advancements in media and technology, and due to changes in individuals’ need for information [1–4]. Consequently, there has been a notable increase in health information available via healthcare providers, the media, printed materials, and the internet. However, the internet remains the most commonly used source [2,5], and its emergence has led to a surge in health information seeking among adults in the United States [1,6,7].

The two most common domains of health information sought are information on health and wellness, including exercise and diet; and information on managing chronic illnesses or disease [4]. One such highly sought disease-related topic is cancer information [3,8]. Cancer is currently the second leading cause of death in the United States, and it is estimated to surpass heart disease as the leading cause of death in the next few years [9]. In 2015, there was more than 1.6 million new cases of cancer, causing almost 600,000 deaths [9]. There are more than 15.5 million individuals in the United States today living with a cancer diagnosis and it is expected to increase to 20 million by 2026 [9].

Cancer diagnosis often triggers the need for more information among cancer patients and their relatives [10]. Thus, there is abundant literature on information seeking among cancer patients, including prevention, lifestyle and risk factors, treatment, prognosis, information needs, physician-patient communication, and new therapies [2,3,10–17]. Previous studies, local, state, and nationally representative, have described health information seeking behavior in general. These studies have concluded that the most common sources of health information include the Internet, health professionals, and family/social support networks [18–21]. Additionally, there is evidence that health information seeking behavior may differ based on health condition and disease type [22,23].

As cancer emerges to become to the leading cause of death in the United States (already the leading cause of death in 22 states) [24], it is important to ascertain whether a cancer diagnosis is an independent predictor of health information seeking behavior. A predictor of whether or not individuals with high-risk of developing cancer reduce risky behavior is their ability and willingness to seek cancer related information [3]. However, less than half of the United States population have sought cancer related health information, and many of these individuals were either frustrated, confused, or doubted the credibility of information received [25]. Additionally, studies have shown that a diagnosis of cancer could induce information avoidance among cancer patients [26,27], and contrary to previous beliefs, information avoidance could be independent of SES and educational level [26,28]. Evidence also shows that health information seeking is mostly in a condition-specific context rather than just random searches [29]. Since the majority of health information seekers seek specific rather than general information [29,30], it is important to understand the differences in health information seeking behavior in the context of cancer as a disease condition.

Understanding the differences in health information seeking behavior based on a cancer diagnosis is grounded in the Planned Risk Information Seeking Model (PRISM) [31], a model that synthesizes constructs from several existing health behavior models, including the Theory of Planned Behavior, and the Theory of Motivated Information Management [31]. PRISM postulates health information seeking as a deliberate or planned behavior, which is a function of a person’s perception of knowledge insufficiency, risk perceptions and responses, and attitudes and beliefs about information seeking [30–32]. Based on the above, our study aimed at understanding differences in health information seeking behavior based on whether information seekers have a cancer diagnosis or not. The psychological effects of a cancer screening, or new cancer diagnosis could range from disbelief, denial, anger, hopelessness and avoidance [33–35]. For example, the decision to practice watchful waiting, active surveillance, or active treatment in patients with prostate cancer requires a careful understanding of risks versus benefits of treatment for each patients [36]. This will no doubt require information seeking from patients and their families. While several theoretical frameworks may help understand cancer related health information seeking behaviors, the PRISM describes the perception of risk of individuals and how this may motivate them to, or not to seek health information.

Elucidating these factors will help ensure that information needs are adequately met by health professionals, policy makers, advocacy groups and other healthcare constituents as well as providing evidence for the type of audience utilizing non-traditional sources of general health information. This is important in view of the shift from a heavily paternalistic paradigm of health communication to a shared-decision making one, with more individuals than ever before seeking health information from alternate sources other than physicians or healthcare providers [20,23,37]. It would also help ensure that reliable information is available in the preferred medium or source of information best utilized by a growing number of health information seekers.

To the best of our knowledge, there has been no nationally representative study that has explored potential differences in information seeking between individuals diagnosed with and without cancer. The goal of this study was to examine if there are differences in health information seeking between respondents diagnosed with and without cancer. We also sought to understand whether there are differences in sources of health information used based on cancer diagnosis.

## Methods

### Data

Analyses are based on data (*n* = 10,774) from Cycles 1–3 (October 2011 to November 2013) of the Health Information National Trends Survey (HINTS) 4 administered by the National Cancer Institute. The HINTS is a nationally-representative probability survey of adults aged 18 or older in the civilian non-institutionalized population of the United States, which assesses usage and trends in health information access and understanding. The algorithm that was used to generate the final weights was the same across cycles and therefore, we appended the cycles and used their associated weights. Details of survey development, design, and methodology have been published elsewhere and are available online [38–40]. All HINTS questionnaires, data, and reports are available at http://hints.cancer.gov/hints4.aspx.

### Dependent variable

The dependent variable was general health information seeking, which was assessed with the screening question: “*Have you ever looked for information about health or medical topics from any source*?” Binary outcomes (yes/no) were generated based on answers to the aforementioned question. Respondents who indicated that they had searched for health or medical information were asked to identify the source they typically use. Responses to the item assessing the source of information were categorized as follows: (1) internet, (2) written materials, (3) health care providers, (4) interpersonal sources, and (5) other. The ‘‘other” category included infrequently mentioned sources including television, radio, cancer organizations, telephone, and specified other.

### Independent variables

The primary independent variable was self-reported cancer diagnosis assessed with the screening question: “*Have you ever been diagnosed as having cancer*?” The response to this question was dichotomized (yes/ no).

### Sociodemographic and behavioral characteristics

Sociodemographic and behavioral characteristics assessed, based on previous literature [5,10] included: Age (18–34, 35–49, 50–64, and 65+); Race/ethnicity (“non-Hispanic White,” “non-Hispanic Black or African American,” “Hispanic,” and “Other” [which includes non-Hispanic American Indian/Alaska Native, Asian, Native Hawaiian/other Pacific Islander, and multiple races mentioned]); Marital status (“married/living as married,” “widowed/divorced/separated,” and “never married”); House income (< $20,000–34,999; $35,000–49,999; $50,000–74,999; $75,000–99,999; and $100,000+); Education (“less than high school,” “high school graduate,” “attended some college,” and “college graduate or higher”) BMI (“normal/underweight,” “overweight,” and “obese”); Health insurance (yes or no); Healthcare provider (yes or no); General health (excellent/very good, good, and fair/poor [combined due to low frequency counts]); Family history of cancer (yes or no); Smoking status (never smoker, former smoker, and current smoker); Nutrition categorized based on standard guidelines recommending at least 5 servings of fruit/vegetables [41] [“less than 5 servings” and “five servings or more]; and Physical activity based on recommendation for at least 30 minutes of moderate physical activity at least 5 days a week [42] [No Physical Activity, 1–4 times/week, and 5–7 times/week].

### Statistical analysis

Analyses were performed using SAS System for Windows (Version 9.4) procedures which incorporate survey sampling weights to account for the complex sampling design used in HINTS (SAS Institute Inc, Cary, NC). In all cases the percentages reported are based on weighted proportions and thus are estimates of the proportion of the entire population having that characteristic. Chi-Square tests (χ^2^) were used to assess associations between cancer status and socio-demographic and behavioral factors, and also between cancer status and information source. In multivariable analysis, a logistic regression model was constructed to evaluate the association between health information seeking and all socio-demographic and behavioral characteristics. We assessed potential collinearity of the covariates with the variance inflation factor (VIF), which is a measure of correlation between pairs of variables [43]. Values of VIF > 10 denote a potentially problematic collinearity within the set of covariates, indicating that these covariates should be removed from model development [44]. None of the VIFs scores in our model was greater than two, which suggests that the variables were not collinear. Statistical significance was determined using a p ≤ 0.05 for all comparisons.

## Results

Table 1 summarizes the study population characteristics stratified by cancer diagnosis status. Almost 80% of respondents were health information seekers. Majority of respondents were non-Hispanic White (66.9%), married or living as married (56.9%), had attained some college education or graduated from college (65.4%), had health insurance (82.1%) and a regular provider (64.0%), and reported good or excellent health (49.2%). In addition, the majority had never smoked (58.7%), consumed five servings or more of fruits and vegetables a day (81.3%), and exercised 1–4 times per week (50.3%). Compared to respondents without a cancer diagnosis, respondents diagnosed with cancer were more likely to be older (over 65 years old), non-Hispanic White, married or living as married, have health insurance, and have a regular provider, but less likely to exercise (p < 0.0001). There were no differences between respondents with and without cancer diagnosis in terms of education, income, BMI, and nutrition.

**Table 1:** Demographic characteristics by cancer status, HINTS 4 Cycles 1–3 (October 2011 to November 2013).

		Diagnosis		
	Overall	Cancer	No Cancer	
	(n = 10,774)	(n = 1,486)	(n = 9,193)	
	n (w%)	w%	w%	*p*-value
**Health Information Seeking**				
Yes	8670 (79.6)	81.2	79.6	
No	2102 (20.4)	18.8	20.4	
**Age**				<.0001
18–34	1537 (29.4)	4.5	31.6	
35–49	2489 (28.1)	12.4	29.5	
50–64	3577 (25.3)	32.2	24.6	
65+	2881 (17.3)	50.9	14.2	
**Gender**				0.0008
Male	4139 (48.5)	42.5	**49.0**	
Female	6382 (51.5)	57.5	**51.0**	
**Race**				<.0001
Non-Hispanic White	6058 (66.9)	82.4	65.6	
Non-Hispanic Black	1493 (10.9)	6.6	11.3	
Hispanic	1483 (14.9)	6.5	15.7	
Other	688 (7.2)	4.5	7.5	
**Marital Status**				<.0001
Married/Living as Married	5607 (56.9)	65.0	56.2	
Divorced/Widowed/Separated	3070 (16.4)	27.2	15.4	
Never Married	1783 (26.7)	7.8	28.4	
**Education**				0.3412
College Graduate	4078 (31.6)	30.4	31.7	
Some College	3157 (33.8)	32.3	34.0	
High School Graduate	2259 (22.6)	23.5	22.5	
Less than High School	1017 (12.0)	13.4	11.8	
**Income level**				0.6180
$100,000 or more	1633 (18.6)	17.5	18.7	
$75,000 to $99,999	1125 (12.4)	13.3	12.3	
$50,000 to $74,999	1564 (17.1)	17.9	17.1	
$35,000 to $49,999	1373 (14.2)	16.0	14.1	
$20,000 to $34,999	1503 (15.6)	14.8	15.2	
$0 to $19,999	2249 (22.1)	20.4	22.1	
**Healthcare Coverage**				<.0001
Yes	9329 (82.1)	94.1	81.1	
No	1318 (17.9)	5.9	18.9	
**Regular Provider**				<.0001
Yes	7349 (64.0)	86.7	62.0	
No	3203 (36.0)	13.3	38.0	
**General Health**				<.0001
Excellent/ Very good	4942 (49.2)	40.8	50.0	
Good	3799 (35.7)	38.2	35.4	
Fair/Poor	1793 (15.1)	21.0	14.6	
**Body Mass Index**				0.8065
Normal/Underweight	3470 (36.2)	35.1	36.4	
Overweight	3561 (33.8)	34.5	33.8	
Obese	3215 (30.0)	30.4	29.8	
**Smoking Status**				<.0001
Never	6112 (58.7)	50.1	59.5	
Former	2795 (22.7)	35.5	21.5	
Current	1687 (18.6)	14.4	19.0	
**Nutrition Status**				0.1282
5 Servings or More	8898 (81.3)	83.6	81.2	
Less than 5 Servings	1876 (18.7)	16.4	18.8	
**Physical Activity**				<.0001
5–7 times per week	2539 (24.1)	25.2	23.9	
1–4 times per wee	5108 (50.3)	43.1	51.1	
No Physical Activity	2947 (25.6)	31.7	25.0	

n—unweighted frequency; w%—weighted percentages

HINTS = Health Information National Trends Survey

Table 2 summarizes our finding on the predictors of general health information seeking overall and among respondents diagnosed with and without cancer. Respondents’ cancer diagnosis status was not associated with general health information seeking (p = 0.6672). Overall gender, education, income, general health, and smoking were significant predictors of health information seeking. Females compared to males were 1.92 (95% CI: 1.53–2.42) times more likely to seek health information as well as respondents who reported fair/poor general health (aOR = 1.81, 95% CI: 1.26–2.60) compared to excellent/very good health. However, respondents who were current smokers compared to never smokers were 30% (95% CI: 0.52–0.95) less likely to seek health information. Similarly, there was a dose-response relationship between health information seeking and education and income. The likelihood of seeking health information decreased across both income and education with the lowest level among those without high school diplomas (aOR = 0.21, 95% CI: 0.14–0.31) compared to those with college degrees as well as those that earn less than $20,000 (aOR = 0.49, 95% CI: 0.32–0.75) compared to over $100,000 income earners.

**Table 2:** Weighted, fully adjusted multivariable logistic regression models predicting health information seeking, overall and stratified by cancer diagnosis status, HINTS 4 Cycles 1–3 (October 2011 to November 2013).

	aOR (95% Confidence Interval)		
	Overall	Cancer Diagnosis	No-Cancer Diagnosis
**Cancer Diagnosis**			
No	Reference	Reference	Reference
Yes	0.93 (0.67, 1.29)	--	--
**Age**			
18–34	Reference	Reference	Reference
35–49	1.10 (0.81, 1.50)	1.49 (0.34, 6.42)	1.10 (0.80, 1.50)
50–64	1.05 (0.78, 1.41)	4.65 (1.18, 18.33)*	0.97 (0.72, 1.31)
65+	0.79 (0.56, 1.10)	3.36 (0.89, 12.61)	0.69 (0.49, 0.97)*
**Gender**			
Male	Reference	Reference	Reference
Female	1.92 (1.53–2.42)**	2.13 (1.27, 3.60)*	1.96 (1.54, 2.50)**
**Race**			
Non-Hispanic White	Reference	Reference	Reference
Non-Hispanic Black	0.76 (0.55, 1.05)	0.80 (0.26, 2.43)	0.76 (0.54, 1.06)
Hispanic	0.63 (0.47, 0.84)*	0.99 (0.40, 2.46)	0.61 (0.45, 0.83)*
Other	0.63 (0.39, 1.02)	1.07 (0.38, 3.01)	0.62 (0.38, 1.02)
**Marital Status**			
Married/Living as Married	Reference	Reference	Reference
Divorced/Widowed/Separated	0.70 (0.56, 0.89)*	0.59 (0.33, 1.04)	0.72 (0.56, 0.93)*
Never Married	0.86 (0.63, 1.17)	0.96 (0.37, 2.48)	0.84 (0.61, 1.16)
**Education**			
College Graduate	Reference	Reference	Reference
Some College	0.53 (0.40, 0.70)**	0.33 (0.15, 0.73)**	0.55 (0.40, 0.74)**
High School Graduate	0.24 (0.18, 0.33)**	0.14 (0.07, 0.32)**	0.25 (0.18, 0.35)**
Less than High School	0.21 (0.14, 0.31)**	0.14 (0.05, 0.38)**	0.21 (0.14, 0.32)**
**Income level**			
$100,000 or more	Reference	Reference	Reference
$75,000 to $99,999	0.52 (0.33, 0.81)*	0.64 (0.23, 1.75)	0.50 (0.31, 0.81)*
$50,000 to $74,999	0.81 (0.55, 1.18)	1.27 (0.47, 3.48)	0.77 (0.52, 1.15)
$35,000 to $49,999	0.59 (0.39, 0.89)*	2.06 (0.72, 5.88)	0.52 (0.34, 0.80)*
$20,000 to $34,999	0.56 (0.37, 0.84)*	1.67 (0.58, 4.85)	0.51 (0.33, 0.78)*
$0 to $19,999	0.49 (0.32, 0.75)*	1.16 (0.40, 3.37)	0.45 (0.29, 0.71)**
**Healthcare Coverage**			
Yes	Reference	Reference	Reference
No	1.05 (0.77, 1.44)	1.10 (0.34, 3.54)	1.06 (0.76, 1.46)
**Regular Provider**			
Yes	Reference	Reference	Reference
No	0.70 (0.55, 0.91)*	0.43 (0.24, 0.76)*	0.71 (0.54, 0.92)*
**General Health**			
Excellent/ Very good	Reference	Reference	Reference
Good	1.25 (0.96, 1.62)	0.77 (0.44, 1.35)	1.30 (0.99, 1.72)
Fair/Poor	1.81 (1.26, 2.60)*	0.96 (0.47, 1.96)	1.89 (1.28, 2.79)*
**Body Mass Index**			
Normal/Underweight	Reference	Reference	Reference
Overweight	1.17 (0.90, 1.51)	1.21 (0.67, 2.20)	1.16 (0.89, 1.53)
Obese	0.99 (0.74, 1.33)	0.71 (0.40, 1.25)	1.01 (0.73, 1.39)
**Smoking Status**			
Never	Reference	Reference	Reference
Former	1.10 (0.86, 1.41)	0.85 (0.48, 1.51)	1.14 (0.87, 1.49)
Current	0.70 (0.52, 0.95)*	0.75 (0.34, 1.66)	0.71 (0.51, 0.97)*
**Nutrition Status**			
5 Servings or More	Reference	Reference	Reference
Less than 5 Servings	0.83 (0.62, 1.11)	1.25 (0.64, 2.44)	0.82 (0.60, 1.12)
**Physical Activity**			
5–7 times per week	Reference	Reference	Reference
1–4 times per wee	1.16 (0.87, 1.53)	1.52 (0.83, 2.79)	1.11 (0.82, 1.50)
No Physical Activity	0.75 (0.55, 1.03)	1.54 (0.79, 3.02)	0.70 (0.50, 0.98)*

**significant at < 0.001 level

*significant at < 0.05 level

aOR = Adjusted Odds Ratio; HINTS = Health Information National Trends Survey

Among individuals diagnosed with cancer, gender, education, and regular provider were significant predictors of health information seeking. Among respondents without cancer diagnosis, age, gender, race/ethnicity, marital status, education, income, regular provider, general health, smoking, and physical activity were significant predictors of health information seeking. After adjusting for covariates, individuals with cancer diagnosis aged 50–64 years were 4.65 (95% CI: 1.18–18.33) times more likely to seek health information whereas this association was not present among respondents without cancer diagnosis. In addition, females compared to males with a cancer diagnosis were 2.13 (95% CI: 1.27–3.60) times more likely to seek health information whereas those without a cancer diagnosis were 1.96 (95% CI: 1.54–2.50) times more likely to seek health information. Moreover, among respondents without a regular healthcare provider, those with a cancer diagnosis were 57% (95% CI: 0.24–0.76) less likely to seek health information while individuals without a cancer diagnosis were 29% (95% CI: 0.54–0.92) less likely to seek health information. Finally, education was an important predictor of health information seeking for both respondents with and without cancer diagnosis. We found that the likelihood of seeking health information declined steadily across education strata. Respondents without a high school diploma compared to those with a college education or higher were 86% (95% CI: 0.05–0.38) less likely to seek health information among those with a cancer diagnosis, and 79% (95% CI: 0.14–0.32) less likely to seek health information among those without a cancer diagnosis.

**Fig 1:**
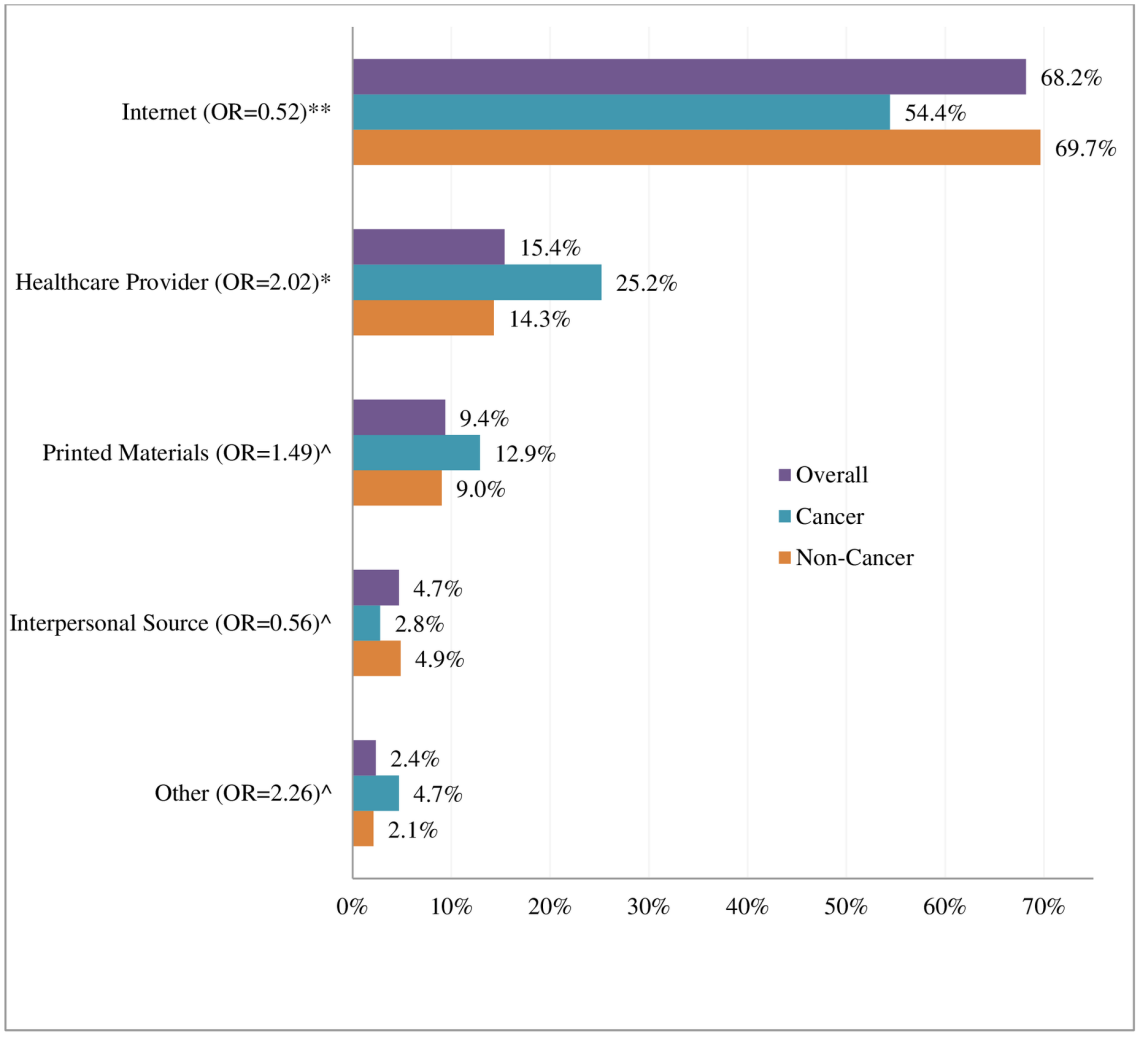
Information seeking sources, HINTS 4 Cycles 1–3 (October 2011 to November 2013). ** Statistically significant at p < 0.05. * Marginally statistically significant at p = 0.05. ^ Not Statistically significant p > 0.05. OR is the odds ratio (Cancer versus non-Cancer) using chi-square tests. HINTS = Health Information National Trends Survey.

Fig 1 summarizes our findings on sources of information used by respondents. Bivariate analyses showed no significant differences in the use of printed materials, interpersonal, and other sources to seek health information. When asked about sources of information used, the top three choices were the same (internet, printed materials, and healthcare provider) irrespective of cancer diagnosis; however they differed in rank orders for the two groups. Respondents without a cancer diagnosis were more likely than those with a diagnosis to use internet (69.7% vs 54.4%; p<0.05) but less likely to use healthcare provider (14.32% vs 25.20%; p = 0.05), and printed materials (9.0% vs 12.9%; p>0.05).

## Discussion

Our study investigated whether having a cancer diagnosis independently predicts health information seeking among adults in the United States. We found no statistically significant difference in health information seeking behavior based on cancer diagnosis. Additionally, while age, gender, education, and having a regular healthcare provider predicted health information seeking among respondents with cancer diagnosis, these same predictors were found among patients without cancer diagnosis as well. However, we found one important difference in health information seeking behavior between these two groups—the source of health information. Our study showed that patients diagnosed with cancer were more likely to use a healthcare provider as a primary source of health information, while patients without a cancer diagnosis were more likely to use the internet. This result is supported by previous findings that show respondents who perceive their health as poor, or who have serious health conditions, are more likely to see a physician as a primary source of health information [5,45]. This does not preclude patients with a cancer diagnosis from using the internet [5]; many patients with cancer diagnoses often use the internet when they want more information about their condition and when they want a second opinion. However, it is important to note that a lot of cancer information on the internet is not peer-reviewed [46], so patients diagnosed with cancer may perceive the need to exercise caution so they have a trusted source of information to meet their cancer information needs.

Potential important reasons exist for the reliance on healthcare providers by respondents diagnosed with cancer for health information. First, cancer diagnoses are usually made and confirmed in a healthcare setting rather than self-diagnosis. Second, a diagnosis of cancer is usually a life-changing event for both patients and family members. Thus, it is plausible that those diagnosed with cancer seek a traditional, trusted source of health information. According to the PRISM, health information seeking is a function of perception of risk as well as of knowledge insufficiency [31]. With the majority of individuals perceiving cancer as a death sentence [47], it is reasonable that cancer patients seek information from a traditional, trusted source of health information such as a physician. Lastly, it may be more convenient for cancer patients to seek information directly from a physician because they may interact with their physician often for other cancer related services.

Our study revealed that females, regardless of cancer diagnosis, were more likely to seek health information compared to males. This result is also consistent with previous findings which identify gender as an important predictor of health information seeking [48],[49–51]. Various physiological, emotional, motivational, cognitive, and personal factors explain, to some extent, the gender difference in health information seeking. In general, men demonstrate low motivation to seek health information [52,53]. Studies also indicate that women are historically more proactive than men in seeking health-related information [2,54–56]. Additionally, Ek (2013) found that women received far more health-related information from interpersonal sources, such as family, friends, and at work than men did [57]. It is important to note that Ek and colleagues did not consider whether females in their study population [57] received information through scanning processes [58], which may have contributed to their findings and explained the gender difference we observed in our study. Nevertheless, the consistent gender difference in health information seeking and the globally persistent gender gap in life expectancy point to a need for promoting gender equality in health by encouraging men to utilize available health information.

In the present study, we found a dose response relationship between respondents’ education and their health information seeking behavior across all categories examined, regardless of cancer diagnosis. The more educated the respondent was, the more likely he/she sought health information. These findings are consistent with previous literature on health-related information seeking, both in the general population and among cancer patients [1,59,60]. The growing quantity and availability of health information requires a need for greater health literacy to obtain and understand information about health in general. Previous research indicates that education plays a key role in determining health literacy, and respondents with lower education are found to have lower health literacy compared to those with higher education [61,62]. Since limited health literacy is associated with worse health outcomes [63–65], targeted intervention programs for those who have less education are important, as they are more likely to be underserved, harder to reach, and easier to miss.

The current study found that respondents without a regular provider were less likely to seek health information regardless of cancer diagnosis status. One possible explanation for this finding could be the role that income may play; previous findings report that economic status is a primary determinant of health information seeking via the internet [66]. Our findings indicate that most of our participants who were not diagnosed with cancer relied on the internet as their information source. Socioeconomic status has been cited as the strongest influence on using the internet for health information [67]. The population we studied had a higher than national income level, so it was not surprising to see that almost 70% of respondents reported using the internet as the primary source of health information. Other socioeconomic factors that affected health information seeking in our study included age and income, with older-aged individuals and lower-income earners being less likely to seek health information. These findings are consistent with another study conducted in a general population [68]. However, Nguyen and colleagues measured specifically online information seeking and not general information seeking behavior, as in our study. Individuals who are older or earn lower income rely on healthcare professionals as their main source of information and therefore get health information only when they see the healthcare professionals [69] compared with the younger and higher-income earners who rely on the internet where health information is ubiquitous [70]. This may explain why older-aged individuals and lower-income earners are less likely to seek health information. Additionally, individuals lower-income earners may prioritize other things like being able to feed their family over seeking health information [71].

### Implications

Knowing the characteristics of patients who are more likely to seek health information based on cancer diagnosis will help allocate resources, programs, and interventions to the appropriate groups. For example, targeting lesser educated individuals is critical because they tend to be underserved and more difficult to reach; this effort would help decrease health disparities related to socioeconomic status. Ensuring the availability of accurate and reliable health information on the Internet [37,46], which was the preferred source of health information in non-cancer diagnosed patients in our study sample, may help ensure accessibility of information in a subgroup of individuals limited by socioeconomically related barriers to health care [72]. In addition, understanding preferred sources of health information is a vital component of patient-centered care. We found that cancer-diagnosed patients preferred information from their health care provider, which suggests that health care providers may need to capitalize on the opportunity to meet unique information needs in the clinical setting when this subgroup is likely to want their health-related inquiries met. Doctors and health care providers who understand their patients’ likely preferred source of information, based on their cancer status, can devote their limited time and resources to providing information that patients will likely pay attention to and utilize. On the other hand, in a study on the general population that also used the HINTS, researchers found that the Internet—via mobile technology—as a preferred source of information influenced the use of and reliance on online health information [73]. If patients are provided information in their preferred source, they may be more likely to feel empowered, participate in treatment decisions, and practice good health and self-care behaviors. These traits may then lead to increased patient satisfaction and better health outcomes [27].

### Strengths and limitations

There are limitations to the current study. First, the cross-sectional design of the HINTS makes the study correlational and prohibits drawing causal inference. Second, variables such as length of time from cancer diagnosis were not assessed because they were unavailable in HINTS. If measured, such variables could have influenced the magnitude and direction of the associations we found. Third, health information seeking was self-reported which could lead to self-report and recall bias. Finally, a single-item measure was used to ascertain our outcome of interest which can lead to measurement error. A multiple item measure could reduce such measurement error. Despite these limitations, the current study has strengths. First, it is one of the first of its kind to stratify a nationally-representative population by cancer status to examine differences in health-information seeking. This is important because health information seeking behavior may be context and disease specific, and patterns are not uniform across populations [30]. In addition, the large sample size we used for analysis provided a powered study wherein we also adjusted for many important variables identified in previous literature as possible confounders.

In conclusion, we described the differences in health information seeking behavior of adults in the United States based on their cancer diagnosis status, and we found that respondents generally seek health information independent of their cancer diagnosis status. However, respondents diagnosed with cancer are more likely to seek information from healthcare provider. With this in mind, it is important that future health information interventions for cancer patients incorporate clear physician-patient communication constructs, since there remains an American population who trusts information from a healthcare provider than the internet. Future studies can examine trends in health-information seeking behavior using various iterations of HINTS data, which is available in multiple cycles.
